# Development of species specific putative miRNA and its target prediction tool in wheat (*Triticum aestivum* L.)

**DOI:** 10.1038/s41598-019-40333-y

**Published:** 2019-03-07

**Authors:** Sarika Jaiswal, M. A. Iquebal, Vasu Arora, Sonia Sheoran, Pradeep Sharma, U. B. Angadi, Vikas Dahiya, Rajender Singh, Ratan Tiwari, G. P. Singh, Anil Rai, Dinesh Kumar

**Affiliations:** 1 Centre for Agricultural Bioinformatics, ICAR-Indian Agricultural Statistics Research Institute, Library Avenue, PUSA, New Delhi, 110012 India; 2 ICAR-Indian Institute of Wheat and Barley Research, Karnal, Haryana 132001 India

## Abstract

MicroRNA are 20–24 nt, non-coding, single stranded molecule regulating traits and stress response. Tissue and time specific expression limits its detection, thus is major challenge in their discovery. Wheat has limited 119 miRNAs in MiRBase due to limitation of conservation based methodology where old and new miRNA genes gets excluded. This is due to origin of hexaploid wheat by three successive hybridization, older AA, BB and younger DD subgenome. Species specific miRNA prediction (SMIRP concept) based on 152 thermodynamic features of training dataset using support vector machine learning approach has improved prediction accuracy to 97.7%. This has been implemented in *TamiRPred* (http://webtom.cabgrid.res.in/tamirpred). We also report highest number of putative miRNA genes (4464) of wheat from whole genome sequence populated in database developed in PHP and MySQL. TamiRPred has predicted 2092 (>45.10%) additional miRNA which was not predicted by miRLocator. Predicted miRNAs have been validated by miRBase, small RNA libraries, secondary structure, degradome dataset, star miRNA and binding sites in wheat coding region. This tool can accelerate miRNA polymorphism discovery to be used in wheat trait improvement. Since it predicts chromosome-wise miRNA genes with their respective physical location thus can be transferred using linked SSR markers. This prediction approach can be used as model even in other polyploid crops.

## Introduction

MicroRNAs (miRNAs) have been identified as important endogenous regulators to various traits and responses against stresses. Since they are single stranded, non-coding, 20–24 nucleotide small RNAs and major post-transcriptional regulators of gene expression, thus their identification and characterization is of great importance^1^. As their expression is spatial over time and tissue, thus it is very difficult to detect them experimentally in any higher eukaryotic organism^2,3^. Moreover, even among the experimentally validated miRNAs, sometimes we may find dubious examples in databases like miRBase and mirTarBase^4^. Since draft wheat genome sequence is available thus chromosome-wise miRNA prediction can be done by *in silico* approach to get location specific miRNA and they can supplement *in vitro* approach for much pragmatic and efficient miRNA discovery^5^. Location specific miRNA can be easily used to discover miRNA polymorphism by designing primers in the flanking regions. MiRNA polymorphism data can be used in association studies and associated miRNA can be transferred in breeding program using linked polymorphic SSR^6^. All these require whole genome based approach for miRNA discovery.

There are large number of tools for miRNA prediction like miRNAfinder^7^, MiRscan^8^ and SSCprofiler^9^. Most of them are based on attributes of miRNA conservation across species. Such approach has been successfully used in predicting miRNA in various species across domain of animal and plants. However, very recently it has been reported that during course of evolution, some oldest miRNAs gets “deleted” and some “younger” miRNAs being less conserved remain unpredicted^10^. Since such events are specific to species in question, thus it would be more holistic approach to predict an “atlas” of all putative microRNAs coded in genomic DNA of a given species with species specific approach^11^.

Majority of the tools are based on machine-learning classification techniques to predict true miRNA such as random forests^12^, hidden Markov models^13^, naive Bayes classifiers^14^ and KNN classifiers^15^ but the most common is support vector machines (SVM)^16^. The appropriate classifier selection, feature extraction, class imbalance correction and training data quality results in improvements both, in terms of sensitivity and specificity^17^. Recent report on Species-specific MIRna Predictors (SMIRP) concept^17^ has shown the importance of species specific training dataset in improvement of prediction accuracy.

Wheat is complex hexaploid genome due to its evolutionary history of successive hybridization of three grass family species, namely, *Triticum ururtu, Aegilops speltoides, Aegilops tauschii* (diploid goat grass) contributing AA,BB and DD genomes, respectively. These hybridization event have happened in timescale of 400 KYA to 8.5 KYA^18^. Since it is a unique combination of genome having oldest sub-genome from tetraploid wheat (*T. turgidum*, AA,BB) and a relatively younger sub genome of diploid goat grass (*Aegilops tauschii*, DD), thus conservation independent method of miRNA discovery is required. Inefficiency of homology based miRNA prediction methods particularly in wheat has already been reported warranting species-specific and clade- specific approach^19^.

Multispecies miRNA database, *miRBase* contains just 119 wheat specific miRNAs. Very limited wheat chromosome-wise miRNA information is available which are confined to chromosomes 4A (68 miRNAs), 5D (55 miRNAs), 1AL (14 miRNA families) and 5A (16 miRNA families)^20^. Previously reported wheat miRNA database by Remita *et al*.^21^, contains 5036 pre-miRNA which is compilation of published miRNAs rather than wheat whole genome assembly based prediction. This was based on transcriptomic data rather than whole genome sequence of wheat. Moreover it contains very limited 5036 pre-miRNAs, which too are confined to abiotic stress conditions and development stages only. Thus more holistic mining of miRNA from entire genome by an improved prediction tool using species specific approach and machine learning is yet to be attempted. This is the first report of extensive mining of putative miRNA genes using chromosome-wise wheat reference genome assembly which catalogues all putative miRNA gene “atlas” over wheat genome independent of trait.

Since wheat genome sequence was not available in most of the previous miRNA publications thus no database is available having its chromosomal/ physical location. Such information is necessary to accelerate the miRNA polymorphism discovery required in crop improvement program. A wheat genome atlas having chromosome-wise miRNAs cataloguing in the form of user-friendly database is not available in any of the previous studies. Moreover this earlier database is not in dynamic mode with options to predict chromosome-wise or user defined miRNA evaluation along with their respective and target site prediction over different chromosomes. Earlier methods which are based on mere conservation of miRNAs across various species but does not include “old” and “younger” miRNAs, thus they predict less number of miRNA genes. Looking at complexity and size of wheat genome (~17 Gb), miRNA prediction methodology needs improvement which is possible by Species specific (SMIRP approach) to overcome the existing limitations. This is the first report having chromosome-wise putative wheat miRNAs in form of a database. Since this tool is well validated by existing small RNA library, secondary structure, star sequences, degradome dataset and binding site predictability over wheat genome, thus it can be used as effective and efficient miRNA discovery tool of polyploid wheat genome.

## Result and Discussion

**Figure 1:**
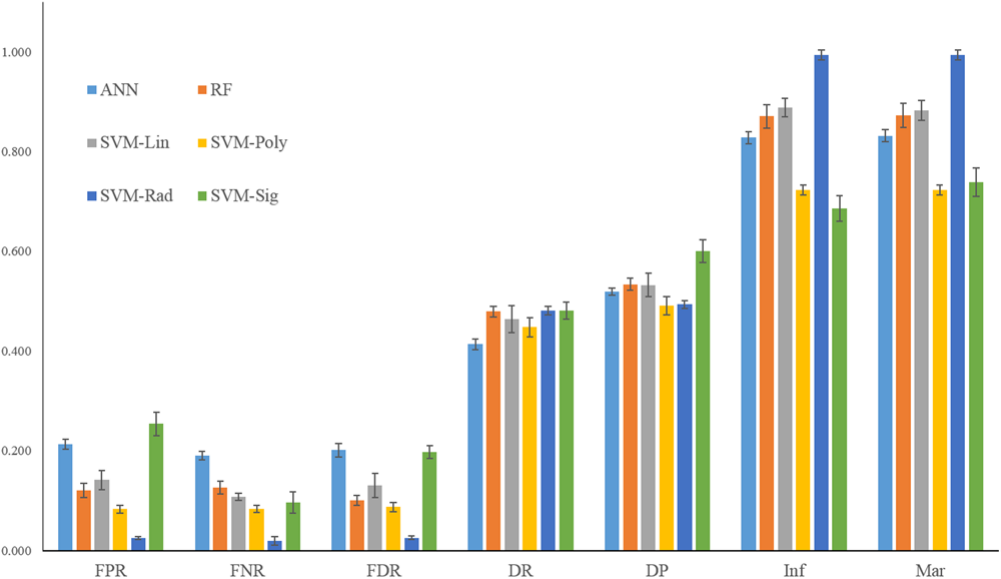
Evaluation measures (FRP:False Positive Rate; FNR:False Negative Rate; FDR:False Discovery Rate; Inf:Informedness; Mar: Markedness) of miRNA prediction models using ANN, RF and SVM methodology.

**Figure 2:**
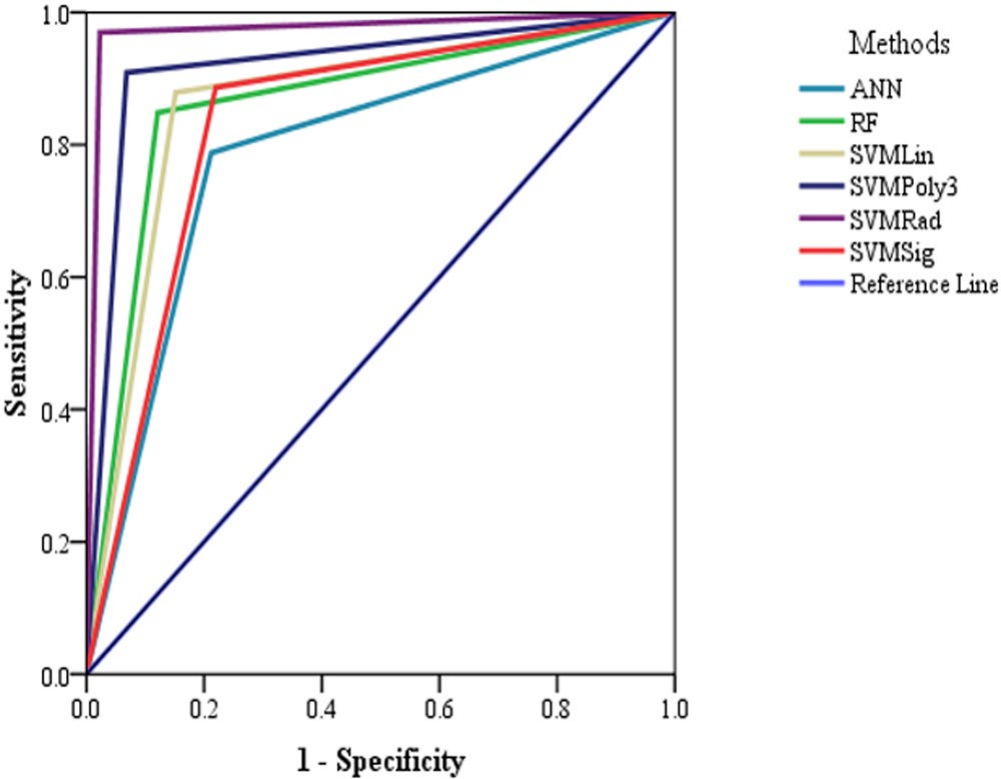
ROC curves for models.

SVM based species specific (SMIRP) methodology was implemented successfully in the web server for putative wheat miRNA along with its target site prediction. Out of 152 features of miRNA, 107 features were found to be significant which were used in model development (Supplementary file 1). Among the machine learning methodologies used in the study, *viz*., ANN, RF and SVM, model developed using SVM-radial basis function (SVM-RBF) was found to have maximum accuracy of 97.7%. The performance of various miRNA prediction models are depicted in Table 1. The various evaluation measures like sensitivity or true positive rate (TPR), specificity or true negative rate (TNR), precision or positive predictive value (PPV), negative predictive value (NPV), fall-out or false positive rate (FPR), false negative rate (FNR), false discovery rate (FDR), accuracy (ACC), F1 score, Matthew’s correlation coefficient (MCC), informedness and markedness discussed in the previous section were adopted to evaluate the models in this study. The model developed using SVM-RBF showed highest informedness while SVM-Sigmoid model showed lowest informedness (Fig. 1). ROC curve for different methodologies used in the study reveals that SVM-RBF classifier performs best with AUC value 0.973 (Table 2 and Fig. 2).

**Table 1:** Performance of the miRNA prediction models using ANN, RF and SVM methodology.

Methods	Se	Sp	PPV	NPV	Accuracy	Precision	Recall	F-measure	MCC
ANN	0.809 ± 0.008	0.787 ± 0.010	0.799 ± 0.014	0.795 ± 0.018	0.797 ± 0.006	0.799 ± 0.014	0.809 ± 0.008	0.803 ± 0.005	0.830 ± 0.012
RF	0.874 ± 0.013	0.880 ± 0.014	0.899 ± 0.010	0.850 ± 0.019	0.877 ± 0.012	0.899 ± 0.010	0.874 ± 0.013	0.886 ± 0.010	0.751 ± 0.024
SVM-Lin	0.892 ± 0.007	0.858 ± 0.20	0.870 ± 0.025	0.877 ± 0.15	0.876 ± 0.009	0.870 ± 0.025	0.892 ± 0.007	0.880 ± 0.013	0.749 ± 0.019
SVM-Poly	0.917 ± 0.007	0.917 ± 0.008	0.913 ± 0.009	0.921 ± 0.004	0.917 ± 0.005	0.913 ± 0.009	0.917 ± 0.007	0.915 ± 0.007	0.834 ± 0.010
**SVM-Rad**	**0.980 ± 0.009**	**0.975** ± **0.003**	**0.974** ± **0.003**	**0.980** ± **0.010**	**0.977** ± **0.005**	**0.974** ± **0.003**	**0.980** ± **0.009**	**0.977** ± **0.005**	**0.955** ± **0.010**
SVM-Sig	0.904 ± 0.021	0.746 ± 0.013	0.803 ± 0.013	0.878 ± 0.023	0.831 ± 0.013	0.803 ± 0.013	0.904 ± 0.021	0.850 ± 0.012	0.665 ± 0.026

**Table 2:** Area Under the Curve of the various methodologies.

Models	Area	Standard Error	Asymptotic 95% Confidence Interval	
			Lower Bound	Upper Bound
ANN	0.788	0.029	0.731	0.845
RF	0.864	0.024	0.816	0.912
SVM-Linear	0.864	0.024	0.816	0.912
SVM-Polynomial3	0.920	0.019	0.883	0.958
SVM-RBF	0.973	0.011	0.951	0.996
SVM-Sigmoid	0.833	0.027	0.781	0.885

This is the first comprehensive report of chromosome-wise miRNAs in wheat which are highest in number also. An atlas of 4464 predicted wheat miRNAs are which are catalogued chromosome-wise are available in the database. Highest (615) and lowest (19) densities of miRNAs were found on chromosome 3B and 4D, respectively (Table 3). Success of SVM based approach without species specific is widely reported in various crop species. Such diverse sets of conserved, non-conserved, and species-specific miRNAs are reported in Jute^22^. Such species specific approach is also reported in animal genome microRNA gene prediction like cattle^23^.

**Table 3:** Comparative analysis of chromosome-wise 5′ mature miRNAs prediction by TamiRPred and miRLocator.

Chromosome Number	No. of mature 5′ miRNAs (Length ≥ 17) predicted by TamiRPred	No. of mature 5′ miRNAs (Length ≥ 17) predicted by miRLocator
1A	384	227
1B	325	175
1D	75	43
2A	416	259
2B	402	221
2D	77	35
3A	147	97
3B	614	347
3D	45	21
4A	280	173
4B	86	39
4D	19	8
5A	141	78
5B	340	211
5D	103	54
6A	204	111
6B	167	96
6D	126	71
7A	256	141
7B	52	23
7D	205	117
Total	4464	2547

Though SVM based methodology has been used successfully in several crops but number of miRNA genes have been limited, for example in case of tomato 522 miRNAs genes were predicted^24^. With species specific, improved training dataset, we have improved the predictability of miRNAs in wheat. Interestingly, our study reveals very high number 4464 which might be due to complex and larger genome size and also due to scanning of older and new putative miRNAs that covers non-conserved regions as well. This is the major advantage of this species specific SMIRP approach^17^.

## Evaluation of TamiRPred with miRLocator for MiRNA prediction

**Figure 3:**
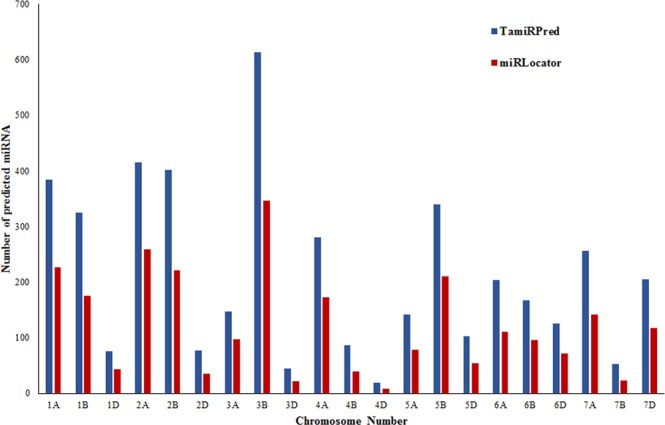
Comparative analysis of chromosome-wise 5′ mature miRNAs prediction by TamiRPred and miRLocator.

The comparative evaluation of developed TamiRPred and existing miRLocator tool revealed significant improvement in miRNA predictability. Out of 4464, nearly half of them could get predicted (2547 pre-miRNA sequences representing 54.90%) and 45.10% (2092 pre-miRNA sequences) remained unpredicted. This demonstrates that by earlier existing tool, miRLocator, a substantial portion of miRNA would have been missed in prediction. Chromosome-wise improved prediction is shown in Table 3 and Fig. 3. It clearly reveals that improved predictability of TamiRPred on each chromosome of entire wheat genome with similar magnitude of miRNA density, proportionate to the chromosomal length of the reference genome assembly used. Highest and lowest number of miRNAs was observed on chromosome 3B and 4D, respectively by both the tools. These results prove the holistic improvement in prediction ability of TamiRPred without any biasness over any of the specific chromosome number. Coincidently, we found highest number of miRNA genes on chromosome 3B which was the longest chromosome in genome assembly used but this is contrary to the widely accepted fact that miRNA density is independent of chromosomal length^25^. Since the genome assembly used is not fully finished, thus it will be too early to conclude anything in terms of miRNA density and chromosomal length.

### Computational validation of predicted miRNA

#### Detection of predicted miRNA in smallRNA library of wheat

Out of total 4464 miRNA predicted sequences, 1906 were found present in smallRNA library of 11 tissues in 158 sets (Table 4). This limited validation in tune of 41% is due to inherent attributes of miRNA genes for their spatial-temporal expression^2,3,26^. Due to this reason it is very difficult to detect all of them experimentally in limited dataset of small RNA libraries. Since this validation has been done with very large set of small RNA libraries of wheat having various tissues like leaf, root, flower, shoot, spike, microspore embryo, seedling with different stages/ timeline as well as biotic and abiotic stress treatments, thus it firmly validates the efficacy of TamiRPred tool.

**Table 4:** Validation of predicted miRNAs in wheat small RNA library.

Sr. No.	BioProject IDs	Number of detected miRNAs in wheat small RNA library
1.	PRJNA218544	1104
2.	PRJNA232120	1138
3.	PRJNA244006	1021
4.	PRJNA297977	1070
5.	PRJNA326902	1024
	Total	5357 (Unique: 1906)

#### Detection of stem-loop structure

**Figure 4:**
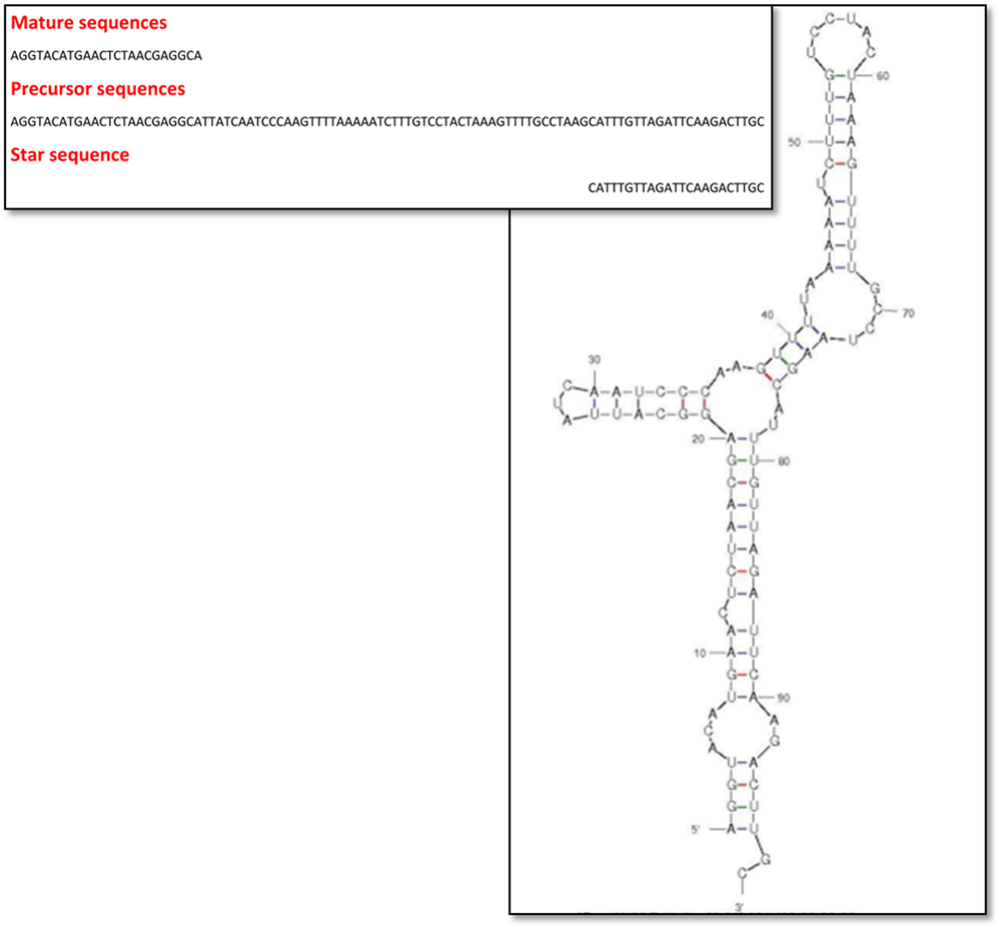
Structure of precursor miRNAs with bulge, stem loop, hairpin and star sequence.

Structure of precursor miRNAs in RNAFold was found with bulge, stem loop, hairpin and star sequence. Mature and star sequences were found in complementarity with permissible gap and penalty (Fig. 4).

#### Detection of homologous miRNA in miRBase

A total of 213 homologous miRNA were found in miRBase. These miRNAs are also called candidate miRNAs. They are present in various crop due to their extensive conservation across different species.

#### Validation of TamiRPred miRNA precursor predictability by star miRNA

Analysis of five small RNA libraries of wheat using miRCat revealed 1105 miRNAs having their star sequences. A total of 594 miRNAs sets having their star sequences also were found matching with that of miRNA predicted by TaMiRPred using wheat genome assembly (Supplementary file 2). This positive validation confirms the predictability of the tool.

#### Validation of TaMiRPred mature miRNA predictability using degradome dataset

TamiRPred predicted 4464 mature miRNAs analysis with available degradome dataset of 953 mature miRNA having its corresponding star miRNA revealed presence of 706 mature miRNAs (Supplementary file 3). This clearly demonstrates that mature miRNA predicted by TaMiRPred actually exists in the tissue in its mature form and existence of star miRNA further confirms their acceptability.

#### Validation by prediction of binding site over wheat coding sequence

TamiRPred successfully predicts binding sites of predicted miRNA. It also has provision for both finding specific target genes as well as user defined/ published miRNA. The approach of cross linking offers significant advantage as it can automatically take care of future updated data. At the moment limited reports are available on miRNA in wheat for different traits related to production, abiotic and abiotic stress. For example, drought and heat^27^, cold stress^28^, wheat powdery mildew^27^, leaf rust^29^, grain development^30^, phosphate use efficiency in *A. tauschii* (D genome progenitor of wheat)^31^, delayed heading time, male sterility^32^ and salinity^33^. Our database can accelerate such type of research by both evaluating the reported miRNA as well as predicting new miRNA from wheat genome along with its biological network with other genes.

All the six different validation approach and their success clearly demonstrates that TamiRPred predicted miRNA genes actually gets expressed. These results further confirms the TamiRPred is better tool to predict miRNA in wheat genome, over existing methods. This successful approach of miRNA predicting using machine learning followed by series of validation in a complex polyploid genome like wheat can be used as a model for other polyploid genomes also.

## Utility of TamiRPred

**Figure 5:**
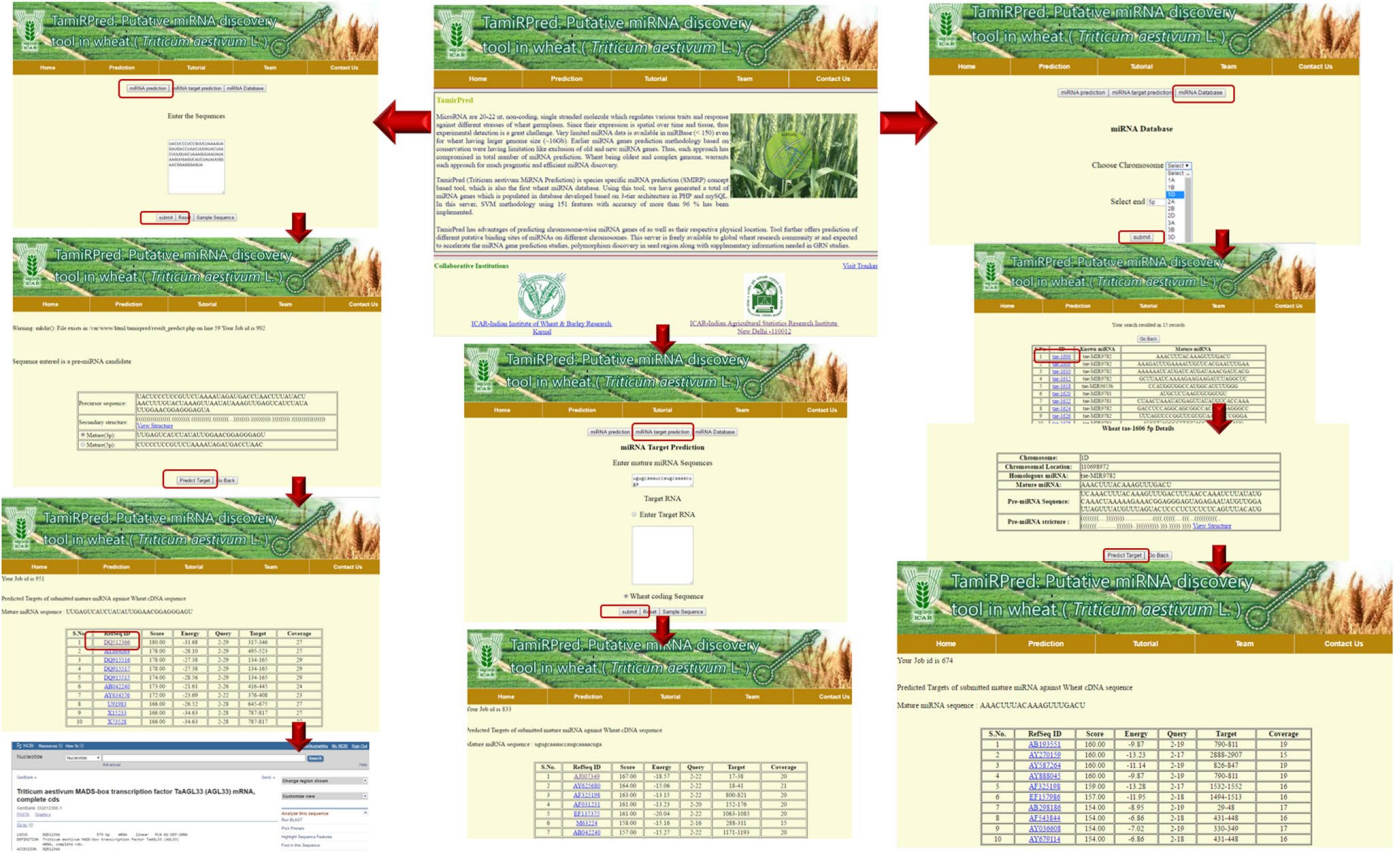
Various search options of TamiRPred.

TamiRPred can be used for three different purposes as a research tool viz., miRNA Prediction, miRNA target prediction and chromosome-wise (location specific) miRNA mining. In long run, it can be used to make secondary database of miRNA polymorphism by targeted SNP discovery in seed region as well as UTR binding region of miRNA. Study has shown that SNPs within 3′ UTR region affects function of miRNA, thus can be a putative candidate gene having very high relevance in genome wide association studies (GWAS) and eQTL studies^34^. Our server can be used to develop miRNA polymorphism database in future which may be another tool for future association studies in wheat improvement program. Such miRNA polymorphism in crop is of high commercial importance for example, in case of maize for trait like drought tolerance, a single miRNA is patented^35^. In case of rice, miRNA polymorphism is reported to be associated with seed length^36^. Since such miRNA polymorphism along with trait association is yet to be initiated in wheat, thus TamiRPred can be pivotal in designing experiment of polymorphism discovery. Since miRNA mediates gene regulation, thus miRNA-based genetic modification technology (miRNA-based GM tech) has potential to contribute in increasing the agricultural productivity by controlling the biotic and abiotic stress tolerance^37^. Figure 5 shows various search options of TamiRPred.

TamiRPred server will be a research tool freely available to global wheat research community for miRNA and its target prediction. This server has multiple use for example, researcher can predict whether a given query sequence of wheat genome fragment or RNASeq is harbouring putative miRNA gene or not. Even an existing or known miRNA can also be used over this server to predict its putative binding site to understand its potential role in regulation of gene expression or gene regulatory network. Researcher can use the server further in order to get chromosome-wise microRNA gene prediction along with its exact physical location. Till now there is no wheat miRNA database giving physical location. This tool can accelerate miRNA polymorphism discovery in seed region of miRNA gene as well as binding site of miRNA. Such miRNA polymorphism has been used for association studies^6^. Besides this knowledge discovery, such tool has immense future utility, especially in miRNA-SSR polymorphism data generation required for genetic diversity analysis, marker-assisted selection and genotyping in wheat. For generating polymorphism data targeted amplicon sequencing can be done by designing the primer over flanking region of miRNA as well as linked SSR loci of a given chromosome/location. Such miRNA-SSR polymorphism discovery using various germplasm accessions has been reported in model leguminous research crop barrel medick (*Medicago*)^38^. For chromosomal location specific miRNA-SSR polymorphism discovery, TaSSRDb (having > 475 thousands SSRs) developed by our group^39^ which is also freely accessible, can be used. Since our tool can be a valuable resource for accelerated polymorphism discovery in wheat genome focusing on miRNA seed and UTR region polymorphism as well as linked SSR polymorphism, thus can be of immense use in molecular breeding for wheat variety improvement.

Present work has two major significance in wheat miRNA research. First, it improves the miRNA predictability due to machine learning approach which is especially needed for complex polypoid genome having sub-genomes of different evolutionary time-scale. This improvement over existing tool is well demonstrated by validating the finding using wheat miRNA library from 11 wheat tissues, presence of star miRNAs and wheat specific degradome dataset. Second, in terms of utility in molecular breeding program for improvement of wheat germplasm. This first miRNA atlas in server mode having chromosome-wise location can be used for miRNA polymorphism discovery required for trait association studies in wheat. In long run, present tool can accelerate the development of wheat miRNA polymorphism database.

## Conclusion

We report here whole genome based first miRNA genes database of wheat based on species specific miRNA prediction (SMIRP concept) with significantly improved predictability over existing method. SVM methodology using 152 features of species specific improved training dataset with accuracy of 97.7% has been successfully implemented in TamiRPred. This tool is freely available to global research community of wheat at http://webtom.cabgrid.res.in/tamirpred/. We have evaluated improved predictability of our tool with respect to existing tool and we report highest 4464 number of putative miRNA genes in wheat. These miRNAs have been confirmed by validation of 2829 miRNAs in 158 small RNA libraries of different wheat tissues. These results have been further validated by binding site prediction data also. Genes of miRNA can be predicted chromosome-wise along with their respective physical location. Such tool can accelerate the miRNA gene prediction studies, polymorphism discovery in seed region along with supplementary information needed in gene regulatory network (GRN) studies. Polymorphic miRNA can be used in association studies and linked SSR markers can be used for marker assisted transferability of selected miRNA in wheat molecular breeding program for germplasm improvement. Since this study reveals improved miRNA prediction accuracy using species specific approach, especially for complex and polyploid genome like wheat, thus can be a model approach in other polyploid species for accelerated miRNA prediction.

## Material and Methods

### Data availability and pre processing

For the present study, complete genome sequence of *T. aestivum* (2n = 6x = 42, AABBDD), was downloaded from Ensembl (ftp://ftp.ensemblgenomes.org/pub/release-30/plants/fasta/triticum_aestivum/cds/). In order to generate species specific training dataset of miRNAs for machine learning approach, all the existing known and published (source cited on webserver) wheat miRNA hairpin precursor and mature miRNA sequences were retrieved. Cd-HIT^40^ was used to remove the repeated miRNA sequences resulting in 439 unique sequences (file separately available on webserver), which were further employed as query sequences for BLAST search against wheat genome available at *Ensembl*. The sensitive BLASTN parameter setting of word-length 7 and E-value cut off as10 was fixed to identify potential miRNA candidates. Top 10 BLAST hit records were extracted and miRNA candidate sequences with <5 mismatches after BLAST parse considering sequence variations against known miRNAs were adopted for further use. A total of 7303 BLAST hits passed this for further analysis.

Scripts were written in perl to extract sequences of 500-nt upstream and 500-nt downstream from these 7303 potential miRNA candidates. These were further fragmented with 90 nucleotide, 100 nucleotide, 110 nucleotide, 120 nucleotide-sliding window with a step of 10 nucleotide, resulting in approximately 3,03,983 fragments (90–120 length with no overlap). Hairpin structure (RNA Secondary structure) as well as Minimum Folding Energy were predicted using RNAfold (Zuker’s Algorithm) from Vienna RNA Package^41^ for all the fragments. After removal of pseudo miRNAs using perl scripts with the parameters (stem length 20–50 nucleotide, GC content 24–82% and MFE −20 to −60), a total of 159052 candidate miRNA precursors were obtained.

MiRNA exists in stem-loop structure due to thermodynamic properties of nucleotides and it varies from sequence to sequence. In order to ensure prediction accuracy of miRNA, its sequence must match with its specific precursor miRNA even in its secondary structure confirming stem and loop structure using RNAFold^42^. If data of thermodynamic features of large number of miRNA training dataset are used by machine learning approach to develop a model, then miRNA can be predicted from any unknown sequence. For each sequence, a total of 152 features used in miPlantPremat^24^ were calculated using perl script, of which 107 significant features were finally employed (Supplementary File 1). The support vector machine model was developed using SVM Light for prediction of precursor miRNAs using 439 known mature wheat miRNA sequences as positive set and 439 negative set. The negative set consisted of the sequences extracted from coding sequences of wheat fulfilling the filters like stem length between 20–50 nucleotide, GC content between 24–82% and MFE between −20 to −60. Model was optimized at the parameter values, g = 0.0001, c = 5, j = 3, t = 2.

Each obtained miRNA precursors were tested in this developed model and after filtering those with positive SVM score, we got 37738 real miRNA precursors. Further, a model was developed after achieving mature miRNAs using software *miRdup*^43^. Total of 4464 mature miRNAs were obtained whose targets were predicted using *miRanda*^44^.

### Support Vector Machine

In order to develop the classification, Artificial Neural Networks (ANNs) with back propagation algorithm have been used earlier^45,46^. This algorithm was found to overfit the model and led to underestimation of actual prediction error, especially in case of small sample size. Further, random forest (RF) classification algorithm, which is combination of Breiman’s “bagging” idea and the random selection of features, introduced independently by Ho (1998)^47^ and Amit and Geman (1997)^48^, was developed by Breiman (2001)^49^ to construct a collection of decision trees with controlled variation. RF too has been observed to overfit for some datasets with noisy classification/regression tasks^50^. In case of small sample size, a nonparametric algorithm, Support Vector Machine (SVM) which was developed by Vapnik (2000)^51^ is quite reliable due to its non-linear optimization property. For small sample, nonlinearity and high dimensional data application, SVM is very popular and promising algorithm in classification. It has ability to handle noise and large input spaces in case of biological analysis^52,53^. This algorithm is based on structural risk minimization (SRM) principle. The kernel function allows the nonlinear mapping of input space to high dimensional feature space which decides the power of SVM and hence is one of the important issues during training. In our study, we used linear, polynomial with degree d, Radial Basis Function (RBF) and sigmoid kernel function^54^ which are expressed as follows:

1 $$K({{\bf{x}}}_{i},{{\bf{x}}}_{j})={{\bf{x}}}_{i}^{T}{{\bf{x}}}_{j}\,({\rm{Linear}}\,{\rm{SVM}})$$

2 $$K({{\bf{x}}}_{i},{{\bf{x}}}_{j})={(\gamma {{\bf{x}}}_{i}^{T}{{\bf{x}}}_{j}+r)}^{d}\,({\rm{Polynomial}}\,{\rm{SVM}}\,{\rm{of}}\,{\rm{degree}}\,{\rm{d}})$$

3 $$K({{\bf{x}}}_{i},{{\bf{x}}}_{j})=\exp \,\{\,-\,\gamma ||{{\bf{x}}}_{i}-{{\bf{x}}}_{j}|{|}^{2}\}\,({\rm{Radial}}\,{\rm{Basis}}\,{\rm{function}}\,{\rm{Kernel}})$$

4 $$K({{\bf{x}}}_{i},{{\bf{x}}}_{j})=\,\tanh \,(\gamma \,{{\bf{x}}}_{i}^{T}{{\bf{x}}}_{j}+r)\,({\rm{Sigmoid}})$$

where $r,d,\,\gamma  > 0$ are the kernel parameters, ${{\bf{x}}}_{i}\in {\Re }^{d},\,(i=1,\,2,\mathrm{...},N)$ are the series of input vectors.

### Five-fold cross validation

We used five-fold cross validation technique^55^ for evaluation of developed models. In five-fold cross validation, the whole dataset is randomly divided into five equal sets. Four among these five sets are used for training and the remaining one is for testing. This is repeated five times so that each set goes under test set. Finally, average of five sets is taken.

### Performance evaluation

The fitted models were evaluated using test data. Following measures for statistical estimation of the accuracy of prediction models were used^56,57^.

### Measure

$${\rm{Sensitivity}}\,{\rm{or}}\,{\rm{true}}\,{\rm{positive}}\,\mathrm{rate}\,({\rm{TPR}})=\frac{{\rm{TP}}}{{\rm{TP}}+{\rm{FN}}}$$

$${\rm{False}}\,{\rm{Discovery}}\,\mathrm{Rate}\,({\rm{FDR}})=\frac{{\rm{FP}}}{{\rm{TP}}+{\rm{FP}}}$$

$${\rm{Specificity}}\,{\rm{or}}\,{\rm{true}}\,{\rm{negative}}\,\mathrm{rate}\,({\rm{TNR}})=\frac{{\rm{TN}}}{{\rm{TN}}+{\rm{FP}}}$$

$$\mathrm{Accuracy}\,({\rm{ACC}})=\frac{{\rm{TP}}+{\rm{TN}}}{{\rm{TP}}+{\rm{FP}}+{\rm{FN}}+{\rm{TN}}}$$

$${\rm{Precision}}\,{\rm{or}}\,{\rm{Positive}}\,{\rm{Predictive}}\,\mathrm{Value}\,({\rm{PPV}})=\frac{{\rm{TP}}}{{\rm{TP}}+{\rm{FP}}}$$

$${\rm{F}}1=\frac{2{\rm{TP}}}{2{\rm{TP}}+{\rm{FP}}+{\rm{FN}}}$$

$${\rm{Negative}}\,{\rm{Predictive}}\,\mathrm{Value}\,({\rm{NPV}})=\frac{{\rm{TN}}}{{\rm{TN}}+{\rm{FN}}}$$

$${\rm{Matthew}}\mbox{'}{\rm{s}}\,{\rm{correlation}}\,\mathrm{coefficient}\,({\rm{MCC}})=\frac{({\rm{TP}}\times {\rm{TN}})-({\rm{FP}}\times {\rm{FN}})}{\sqrt{({\rm{TP}}+{\rm{FP}})({\rm{TP}}+{\rm{FN}})({\rm{TN}}+{\rm{FP}})({\rm{TN}}+{\rm{FN}})}}$$

$${\rm{Fall}}-{\rm{out}}\,{\rm{or}}\,{\rm{False}}\,{\rm{Positive}}\,\mathrm{Rate}\,({\rm{FPR}})=\frac{{\rm{FP}}}{{\rm{FP}}+{\rm{TN}}}$$

$${\rm{Informedness}}={\rm{TPR}}+{\rm{SPC}}-1$$

$${\rm{False}}\,{\rm{Negative}}\,\mathrm{Rate}\,({\rm{FNR}})=\frac{{\rm{FN}}}{{\rm{TP}}+{\rm{FN}}}$$

$${\rm{Markedness}}={\rm{PPV}}+{\rm{NPV}}-1$$

For our study,

True positive (*TP*): The number of precursor miRNAs correctly predicted as precursor miRNAs

True negative (*TN*): The number of non-precursor miRNAs correctly predicted as non- precursor miRNAs

False negative (*FN*): The number of precursor miRNAs incorrectly predicted as non- precursor miRNAs

False positive (*FP*): The number of non-precursor miRNAs incorrectly predicted as precursor miRNAs.

Area under receiving operating characteristic curve (AUC-ROC) was further used to measure predictive ability. For given false positive rate (α) and true positive rate (1 − β) at different threshold values, the AUC-ROC was computed as:

5 $$AUC=\sum _{i}\{(1-{\beta }_{i}{\rm{\Delta }}\alpha )+\frac{1}{2}[{\rm{\Delta }}(1-\beta ){\rm{\Delta }}\alpha ]\}$$

where ${\rm{\Delta }}(1-\beta )=(1-{\beta }_{i})-(1-{\beta }_{i-1})\,\,{\rm{and}}\,\,{\rm{\Delta }}\alpha ={\alpha }_{i}-{\alpha }_{i-1}$ and *i* = 1, 2, …, *m* (number of test data points)^58^. Computer program was developed using R programming language to compute the values of these performance measures.

### Computational validation of predicted miRNA

#### Detection of predicted miRNA in smallRNA library of wheat

All predicted miRNA sequences by TaMiRPred were validated in small RNA library of wheat using public domain data. A total of five BioProjects having 158 sets of small RNA libraries were obtained from NCBI, namely, PRJNA218544, PRJNA232120, PRJNA244006, PRJNA297977 and PRJNA326902 representing eleven wheat tissues. Blastn analysis was used to detect their presence with stringent parameters (identity = 100%, coverage = 100%, mismatch = 0 and gaps = 0).

#### Detection of stem-loop structure

Predicted miRNA were further evaluated for existence of stem-loop structure using RNAFold tool for shape. Manually nucleotide sequence were checked for presence of mature miRNA along with its complementary star sequence within limits of permissible gap and penalty.

#### Detection of homologous miRNA in miRBase

All predicted miRNA of wheat based on wheat genome assembly were searched for presence of homologous miRNA in other crops using miRBase^59^.

#### Validation of TaMirPred miRNA precursor predictability by star miRNA

It is necessary to rule out the presence of false positive among predicted putative novel miRNA, which are not having any homology in miRBase. Since presence of star sequence is mandatory to accept them as truly existing novel miRNA, thus star sequence based validation was also carried out. Available smallRNA library was analysed by MiRCat to obtain predicted miRNA along with their respective star sequence. MiRCat based miRNA sequence having their star sequences were matched with miRNAs predicted by our tool, TaMiRPred.

#### Validation of TaMiRPred mature miRNA predictability using degradome dataset

In silico mature miRNA predictability of TaMiRPred can be revalidated by presence of mature miRNA *in vitro*. Public domain available wheat degradome dataset can be used for this validation. Mining of such degradome dataset was done and TaMiRPred based predicted mature miRNA were matched using PERL script.

#### Validation by prediction of binding site over wheat coding sequence

**Figure 6:**
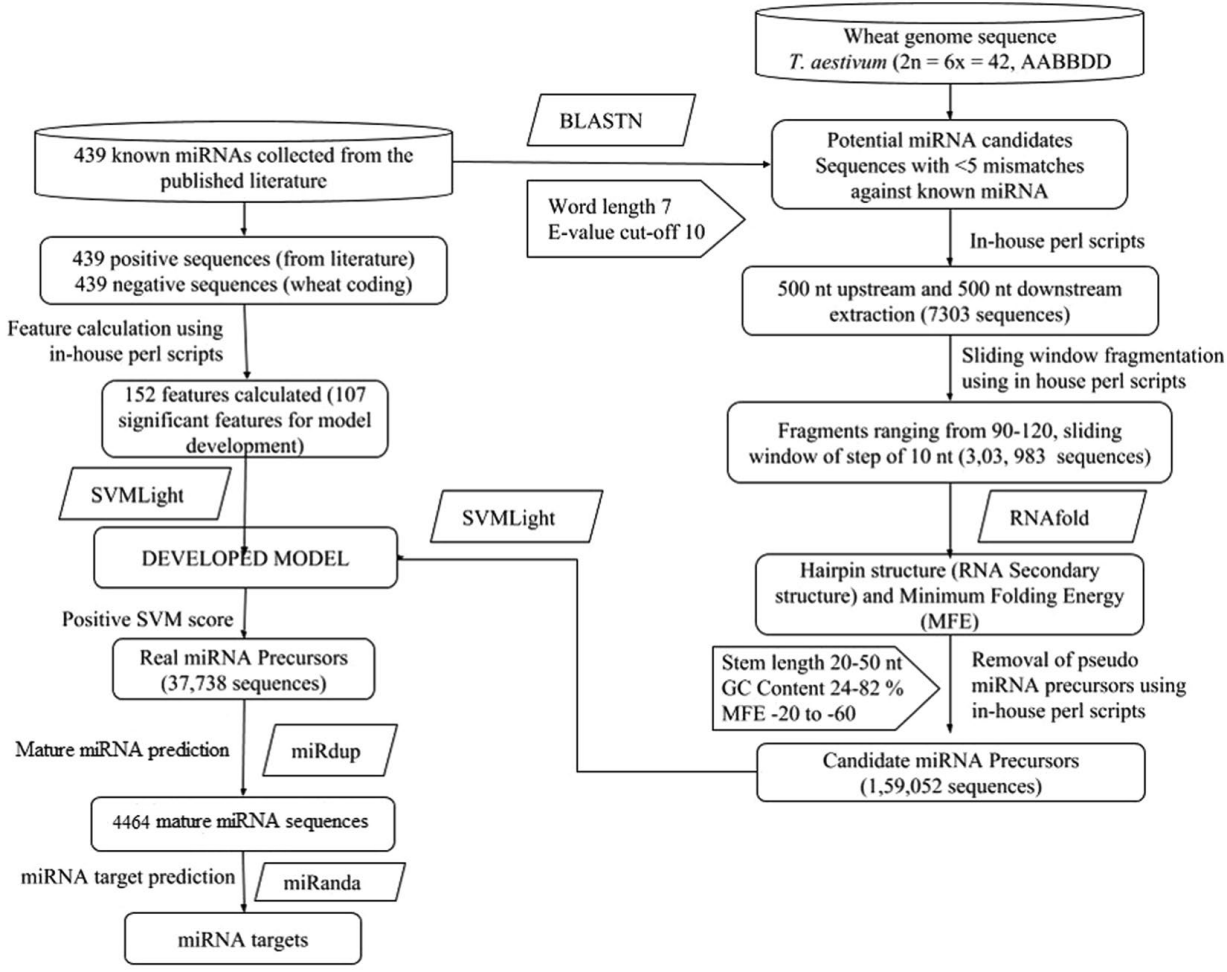
Workflow of wheat miRNA and its target prediction.

Provision has been made to predict binding site of each and every predicted miRNA. miRanda software^60^ has been integrated at the backend along with wheat coding sequence. The output of Miranda gets parsed using perl scripts to generate wider information from NCBI. The workflow of wheat miRNA and its target prediction is illustrated in Fig. 6.

### Database architecture and web implementation

Wheat miRNA server is developed in Apache, PHP and MySQL database. It is a relational database with “three-tier architecture” with a client, middle and database tier. The best model for miRNA prediction was implemented and made available at http://webtom.cabgrid.res.in/tamirpred/. The server has been developed using CGI-Perl script, Hyper Text Markup Language (HTML) and Java Scripts to make it more user-friendly and launched using open source web server software program, Apache. To develop an efficient browsing system, architecture has been enabled with PHP scripts, which provides faster integration and query based searches to the users. Wheat miRNA server has been developed using Apache version 2.2.15, PHP version 5.3.3 and MySQL version 5.1.52 database along with Java version 1.6.0.22. For the prediction of mature miRNA sequences, software miRdup^43^ has been integrated at the backend. For target prediction, miRanda software^44^ has been integrated. The miRNA database has been developed using MySQL and linked to the webserver with the help of open source scripting language PHP and HTML.

This tool can easily be used for miRNA Prediction with their specific chromosomal location as well as their target site over coding regions of wheat genome. For the prediction of new miRNA, user has to give the sequence of interest (query sequence). If the miRNA is predicted in query sequence, then it gives: mature miRNA sequence with 3′ and 5′ end, dot-bracket notation, minimum folding energy, hairpin loop structure. Further, such predicted miRNAs can be analysed in its respective target genes. User can also evaluate any published miRNA for its respective target genes in wheat genome or any transcript can be evaluated whether it is having binding site in its UTR region for any miRNA. This binding site prediction of miRNA has been implemented in TamiRPred using wheat coding sequence for degradome analysis. It has major advantage that it takes care of all existing as well as future wheat mRNA sequence in NCBI. Predicted target sequence represents top ten hits along with the score, energy, query coverage, and target location on gene. User can obtain specific target genes against each miRNA through Refseq link that redirects to its corresponding RefSeq ID. In this miRNA database, user can also use search option to get chromosome-wise miRNA along with the option of 5′ and 3′ end. Further, targeted genes can also be predicted for each of these miRNA.

### Evaluation of TamiRPred with miRLocator for MiRNA prediction

Evaluation of TamiRPred was done by its predicted 4464 pre-miRNA sequences whose mature 5′ miRNA was having length upto 24 nucleotides. These predicted pre-miRNA sequences were taken as query input to predict mature miRNA sequence using existing miRLocator tool^61^.

## Supplementary information

Supplementary File 2

Supplementary File 3

Supplementary file 1

## References

[1] Bartel DP (2004). MicroRNAs: genomics, biogenesis, mechanism, and function. Cell.

[2] Wu Y, Wei B, Liu H, Li T, Rayner S (2011). MiRPara: a SVM-based software tool for prediction of most probable microRNA coding regions in genome scale sequences. BMC Bioinformatics.

[3] Ritchie W, Gao D, Rasko JE (2012). Defining and providing robust controls for microRNA prediction. Bioinformatics.

[4] Saçar MD, Hamzeiy H, Allmer J (2013). Can MiRBase provide positive data for machine learning for the detection of MiRNA hairpins?. Journal of Integrative Bioinformatics.

[5] Khalifa, W., Yousef, M., Demirci, M. D. S., & Allmer, J. The impact of feature selection on one and two-class classification performance for plant microRNAs. *Peer J*. **4**, 10.7717/peerj.2135 (2016). 10.7717/peerj.2135 PMC4924126 27366641

[6] Ziebarth JD, Bhattacharya A, Chen A, Cui Y (2011). PolymiRTS Database 2.0: linking polymorphisms in microRNA target sites with human diseases and complex traits. Nucleic acids research.

[7] Huang T-H (2007). MiRFinder: an improved approach and software implementation for genome-wide fast microRNA precursor scans. BMC Bioinformatics.

[8] Lim LP (2003). The microRNAs of Caenorhabditis elegans. Genes & Development.

[9] Oulas A (2009). Prediction of novel microRNA genes in cancer-associated genomic regions–a combined computational and experimental approach. Nucleic Acids Research.

[10] Nozawa M, Miura S, Nei M (2012). Origins and evolution of microRNA genes in plant species. Genome biology and evolution.

[11] Evers M, Huttner M, Dueck A, Meister G, Engelmann JC (2015). miRA: adaptable novel miRNA identification in plants using small RNA sequencing data. BMC bioinformatics.

[12] Jiang P (2007). MiPred: classification of real and pseudo microRNA precursors using random forest prediction model with combined features. Nucleic acids research.

[13] Kadri S, Hinman V, Benos PV (2009). HHMMiR: efficient de novo prediction of microRNAs using hierarchical hidden Markov models. BMC bioinformatics.

[14] Yousef M (2006). Combining multi-species genomic data for microRNA identification using a Naive Bayes classifier. Bioinformatics.

[15] Lertampaiporn S, Thammarongtham C, Nukoolkit C, Kaewkamnerdpong B, Ruengjitchatchawalya M (2013). Heterogeneous ensemble approach with discriminative features and modified-SMOTEbagging for pre-miRNA classification. Nucleic acids research.

[16] Ding J, Zhou S, Guan J (2010). MiRenSVM: towards better prediction of microRNA precursors using an ensemble SVM classifier with multi-loop features. BMC bioinformatics.

[17] Peace, R. J., Biggar, K. K., Storey, K. B. & Green, J. R. A framework for improving microRNA prediction in non-human genomes. *Nucleic acids research*, **43**(20), 10.1093/nar/gkv698 (2015). 10.1093/nar/gkv698 PMC4787757 26163062

[18] Liu B (2009). Rapid genomic changes in polyploid wheat and related species: implications for genome evolution and genetic improvement. Journal of Genetics and Genomics.

[19] Agharbaoui Z (2015). An integrative approach to identify hexaploid wheat miRNAome associated with development and tolerance to abiotic stress. BMC genomics.

[20] Budak H, Khan Z, Kantar M (2015). History and current status of wheat miRNAs using next-generation sequencing and their roles in development and stress. Briefings in functional genomic.

[21] Remita MA (2016). A novel comprehensive wheat miRNA database, including related bioinformatics software. Current Plant Biology.

[22] Islam, M. T., Ferdous, A. S., Najnin, R. A., Sarker, S. K. & Khan, H. High-throughput sequencing reveals diverse sets of conserved, nonconserved, and species-specific miRNAs in jute. *International journal of genomics*, **2015** (2015). 10.1155/2015/125048 PMC4378336 25861616

[23] Sadeghi B, Ahmadi H, Azimzadeh‐Jamalkandi S, Nassiri MR, Masoudi‐Nejad A (2014). BosFinder: a novell pre‐microRNA gene prediction algorithm in *Bos taurus*. Animal genetics.

[24] Meng J, Liu D, Sun C, Luan Y (2014). Prediction of plant pre-microRNAs and their microRNAs in genome-scale sequences using structure-sequence features and support vector machine. BMC bioinformatics.

[25] Ghorai A, Ghosh U (2014). miRNA gene counts in chromosomes vary widely in a species and biogenesis of miRNA largely depends on transcription or post-transcriptional processing of coding genes. Frontiers in genetics.

[26] Lai X, Wolkenhauer O, Vera J (2016). Understanding microRNA-mediated gene regulatory networks through mathematical modelling. Nucleic Acids Research..

[27] Xin M (2010). Diverse set of microRNAs are responsive to powdery mildew infection and heat stress in wheat (Triticum aestivum L.). BMC plant biology.

[28] Tang Z (2012). Uncovering small RNA-mediated responses to cold stress in a wheat thermosensitive genic male-sterile line by deep sequencing. Plant physiology.

[29] Kumar, D. *et al*. Discovery of novel leaf rust responsive microRNAs in wheat and prediction of their target genes. *Journal of nucleic acids*, **2014** (2014). 10.1155/2014/570176 PMC4144313 25180085

[30] Sun F (2014). Whole-genome discovery of miRNAs and their targets in wheat (Triticum aestivum L.). BMC plant biology.

[31] Jia J (2013). Aegilops tauschii draft genome sequence reveals a gene repertoire for wheat adaptation. Nature.

[32] Wang Y (2012). TamiR159 directed wheat TaGAMYB cleavage and its involvement in anther development and heat response. PloS one.

[33] Feng H (2013). Target of tae-miR408, a chemocyanin-like protein gene (TaCLP1), plays positive roles in wheat response to high-salinity, heavy cupric stress and stripe rust. Plant molecular biology.

[34] Liu C (2012). MirSNP, a database of polymorphisms altering miRNA target sites, identifies miRNA-related SNPs in GWAS SNPs and eQTLs. BMC genomics.

[35] Skalla, D. W., Joseph, D. C. V., Yu, J. K., Wang, D. & Lu, J. U.S. Patent Application No. 15/057, 516 (2016).

[36] Wang C (2013). Loop nucleotide polymorphism in a putative miRNA precursor associated with seed length in rice (Oryza sativa L.). Int J Biol Sci..

[37] Zhou M, Luo H (2013). MicroRNA-mediated gene regulation: potential applications for plant genetic engineering. Plant molecular biology.

[38] Min X (2017). Genome-Wide Development of MicroRNA-Based SSR Markers in Medicago truncatula with Their Transferability Analysis and Utilization in Related Legume Species. International journal of molecular sciences.

[39] Jaiswal, S. *et al*. Putative microsatellite DNA marker-based wheat genomic resource for varietal improvement and management. *Frontiers in plant science* **8** (2017). 10.3389/fpls.2017.02009 PMC5712362 29234333

[40] Li W, Jaroszewski L, Godzik A (2001). Clustering of highly homologous sequences to reduce the size of large protein databases. Bioinformatics.

[41] Lorenz R (2011). ViennaRNA Package 2.0. Algorithms for Molecular Biology.

[42] Ragupathy R (2016). Deep sequencing of wheat sRNA transcriptome reveals distinct temporal expression pattern of miRNAs in response to heat, light and UV. Scientific reports.

[43] Leclercq M, Diallo AB, Blanchette M (2013). Computational prediction of the localization of microRNAs within their pre-miRNA. Nucleic Acids Research.

[44] Betel D, Wilson M, Gabow A, Marks DS, Sander C (2008). The microRNA. org resource: targets and expression. Nucleic acids research.

[45] Cheng B, Titterington DM (1994). Neural networks: A review from a statistical perspective. Statistical science.

[46] Shukla RP, Tripathi KC, Pandey AC, Das IML (2011). Prediction of Indian summer monsoon rainfall using Niño indices: a neural network approach. Atmospheric Research.

[47] Ho TK (1998). The random subspace method for constructing decision forests. IEEE transactions on pattern analysis and machine intelligence.

[48] Amit Y, Geman D (1997). Shape quantization and recognition with randomized trees. Neural computation.

[49] Breiman L (2001). Random forests. Machine learning.

[50] Segal, M. R. Machine learning benchmarks and random forest regression. *Center for Bioinformatics & Molecular Biostatistic* (2004).

[51] Vapnik, V. The nature of statistical learning theory. Springer science & business media (2000).

[52] Brown MP (2000). Knowledge-based analysis of microarray gene expression data by using support vector machines. Proceedings of the National Academy of Sciences.

[53] Ding CH, Dubchak I (2001). Multi-class protein fold recognition using support vector machines and neural networks. Bioinformatics.

[54] Cristianini, N. & Shawe-Taylor, J. *An Introduction to Support Vector Machines and other Kernel-based Learning methods*. (Cambridge University Press, U.K 2000).

[55] Efron B (1983). Estimating the error rate of a prediction rule: improvement on cross-validation. Journal of the American Statistical Association.

[56] Fawcett T (2006). An introduction to ROC analysis. Pattern recognition letters.

[57] Powers DMW (2011). Evaluation: From Precision, Recall and F-Measure to ROC, Informedness, Markedness and Correlation. J. of Mach. Learn. Techn..

[58] Bradley AP (1997). The use of the area under the ROC curve in the evaluation of machine learning algorithms. Pattern recognition.

[59] Meyers BC (2008). Criteria for annotation of plant MicroRNAs. Plant Cell.

[60] John B (2005). Human MicroRNA Targets. PLoS Biolog..

[61] Cui H, Zhai J, Ma C (2015). miRLocator: Machine Learning-Based Prediction of Mature MicroRNAs within Plant Pre-miRNA Sequences. PLoS One..

