# Supplementary material for: Development of species specific putative miRNA and its target prediction tool in wheat (Triticum aestivum L.)
Source: Sci Rep. 2019 Mar 7;9:3790. doi: 10.1038/s41598-019-40333-y (PMC6405928; doi:10.1038/s41598-019-40333-y)
Supplement: Supplementary file 3 — Supplementary file 1 [file 41598_2019_40333_MOESM3_ESM.docx]

**Development of species specific putative miRNA and its target prediction tool in wheat (*Triticum aestivum* L.)**

Sarika Jaiswal^1#^, M A Iquebal^1#^, Vasu Arora^1^, Sonia Sheoran^2^, Pradeep Sharma^2^, U B Angadi^2^, Vikas Dahiya^1^, Rajender Singh^2^, Ratan Tiwari^2^, G P Singh^2^, Anil Rai^1^, Dinesh Kumar^1^*

^1^Centre for Agricultural Bioinformatics, ICAR-Indian Agricultural Statistics Research Institute, Library Avenue, PUSA, New Delhi-110012, INDIA

^2^ICAR-IIWBR, Karnal, Haryana-132001, INDIA

**Supplementary file 1.** List of 107 significant features used in model development

| **Feature** | **Chi-square** | **p-value** | **Feature** | **Chi-square** | **p-value** | **Feature** | **Chi-square** | **p-value** |
| --- | --- | --- | --- | --- | --- | --- | --- | --- |
| **MFE1** | 184.27 | 0.00 | **triplet_begin_32** | 53.07 | 0.00 | **%CG** | 26.54 | 0.00 |
| **dP** | 150.86 | 0.00 | **triplet_end_8** | 47.80 | 0.00 | **triplet_begin_10** | 14.18 | 0.00 |
| **dQ** | 110.93 | 0.00 | **%UA** | 50.21 | 0.00 | **dH** | 28.02 | 0.00 |
| **dD** | 102.93 | 0.00 | **Freq** | 48.65 | 0.00 | **triplet_end_10** | 10.69 | 0.00 |
| **zG** | 166.70 | 0.00 | **triplet_full_1** | 49.24 | 0.00 | **triplet_begin_14** | 13.35 | 0.00 |
| **zP** | 154.81 | 0.00 | **triplet_full_9** | 46.49 | 0.00 | **triplet_full_12** | 25.43 | 0.00 |
| **zQ** | 107.96 | 0.00 | **triplet_begin_17** | 38.29 | 0.00 | **triplet_full_25** | 23.02 | 0.00 |
| **zD** | 110.89 | 0.00 | **triplet_end_1** | 40.18 | 0.00 | **dS** | 24.30 | 0.00 |
| **MFE3** | 113.28 | 0.00 | **dS/L** | 51.58 | 0.00 | **%GA** | 25.70 | 0.00 |
| **Tm** | 99.59 | 0.00 | **%UU** | 45.34 | 0.00 | **triplet_full_16** | 25.61 | 0.00 |
| **au/L** | 114.56 | 0.00 | **triplet_end_2** | 31.19 | 0.00 | **triplet_end_21** | 12.04 | 0.00 |
| **bp/stem** | 136.97 | 0.00 | **MFE6** | 48.27 | 0.00 | **triplet_begin_18** | 8.91 | 0.00 |
| **avg_mis_num** | 121.81 | 0.00 | **MFE4** | 48.25 | 0.00 | **triplet_end_14** | 11.42 | 0.00 |
| **triplet_full_8** | 120.00 | 0.00 | **%AU** | 42.27 | 0.00 | **gc/L** | 24.44 | 0.00 |
| **triplet_full_32** | 105.98 | 0.00 | **triplet_end_5** | 29.07 | 0.00 | **triplet_begin_2** | 11.21 | 0.00 |
| **MFE2** | 92.53 | 0.00 | **%GG** | 43.22 | 0.00 | **triplet_full_23** | 22.33 | 0.00 |
| **dG** | 96.38 | 0.00 | **triplet_full_15** | 40.83 | 0.00 | **%UG** | 23.72 | 0.00 |
| **zF** | 97.08 | 0.00 | **%G+C** | 42.68 | 0.00 | **triplet_begin_13** | 10.57 | 0.01 |
| **mis_num_end** | 89.80 | 0.00 | **triplet_full_14** | 34.64 | 0.00 | **triplet_full_30** | 17.87 | 0.01 |
| **NEFE** | 91.19 | 0.00 | **triplet_full_21** | 34.09 | 0.00 | **triplet_begin_23** | 11.84 | 0.01 |
| **au/stem** | 81.95 | 0.00 | **triplet_full_20** | 38.16 | 0.00 | **triplet_full_22** | 17.33 | 0.01 |
| **triplet_full_17** | 80.75 | 0.00 | **triplet_full_7** | 33.32 | 0.00 | **MFE9** | 17.19 | 0.01 |
| **Tm/L** | 84.20 | 0.00 | **triplet_full_13** | 31.19 | 0.00 | **triplet_end_18** | 6.75 | 0.01 |
| **Div** | 72.18 | 0.00 | **triplet_begin_9** | 22.92 | 0.00 | **%CC** | 19.64 | 0.01 |
| **mis_num_begin** | 69.67 | 0.00 | **triplet_full_10** | 29.71 | 0.00 | **triplet_full_24** | 20.37 | 0.02 |
| **triplet_begin_8** | 61.55 | 0.00 | **triplet_begin_21** | 19.69 | 0.00 | **triplet_begin_19** | 5.14 | 0.02 |
| **triplet_full_18** | 61.69 | 0.00 | **triplet_full_11** | 27.09 | 0.00 | **triplet_end_24** | 14.57 | 0.02 |
| **triplet_full_5** | 63.68 | 0.00 | **triplet_end_25** | 24.80 | 0.00 | **MFE5** | 16.04 | 0.02 |
| **Diff** | 65.05 | 0.00 | **%GC** | 32.05 | 0.00 | **triplet_begin_5** | 6.27 | 0.04 |
| **dH/L** | 63.50 | 0.00 | **%AG** | 33.72 | 0.00 | **triplet_end_15** | 8.10 | 0.04 |
| **triplet_full_2** | 58.03 | 0.00 | **triplet_end_20** | 20.15 | 0.00 | **triplet_begin_25** | 7.97 | 0.05 |
| **dF** | 49.75 | 0.00 | **triplet_begin_1** | 22.15 | 0.00 | **%CA** | 17.13 | 0.05 |
| **triplet_end_32** | 55.02 | 0.00 | **triplet_end_9** | 19.76 | 0.00 | **triplet_end_16** | 11.20 | 0.05 |
| **triplet_full_19** | 51.86 | 0.00 | **triplet_full_4** | 28.24 | 0.00 | **triplet_full_31** | 14.11 | 0.05 |
| **triplet_end_17** | 45.93 | 0.00 | **triplet_end_7** | 16.63 | 0.00 | **triplet_full_27** | 7.64 | 0.05 |
| **gc/stem** | 56.62 | 0.00 | **triplet_begin_12** | 16.18 | 0.00 |  |  |  |
